# Parity, Age at First Birth, and Risk of Death from Bladder Cancer: A Population-Based Cohort Study in Taiwan

**DOI:** 10.3390/ijerph13121197

**Published:** 2016-12-02

**Authors:** Hui-Fen Chiu, Brian K. Chen, Chun-Yuh Yang

**Affiliations:** 1 Department of Pharmacology, College of Medicine, Kaohsiung Medical University, Kaohsiung 80708, Taiwan; chiu358@yahoo.com.tw; 2 Department of Health Services Policy and Management, Arnold School of Public Health, University of South Carolina, Columbia, SC 29201, USA; BCHEN@mailbox.sc.edu; 3 Department of Public Health, College of Health Sciences, Kaohsiung Medical University, Kaohsiung 80708, Taiwan; 4 Division of Environmental Health and Occupational Medicine, National Health Research Institute, Miaoli 35053, Taiwan

**Keywords:** bladder cancer, parity, mortality, cohort study, Taiwan

## Abstract

The evidence is limited on the relationship between reproductive factors and bladder cancer (BC). We studied 1,292,462 women who had a first and singleton delivery between 1 January 1978 and 31 December 1987. Each woman in the study cohort was tracked from their first childbirth to 31 December 2009. Vital status of the women was determined by crosswalking records with a computerized mortality database. We used Cox proportional hazard regression models to estimate the hazard ratios (HRs) of death from BC associated with maternal age at first birth and parity. The data showed 63 BC deaths during 34,980,246 person-years of follow-up. BC mortality rate was 0.90 cases for every 100,000 person-years. Compared with women who gave birth under the age of 23, the adjusted HR was 1.24 (95% confidence interval (CI) = 0.66–2.35) for women who gave birth between age 23 and 26 and 2.30 (95% CI = 1.21–4.39) for women who gave birth over the age of 26. Increasing age at first birth (*p* for trend = 0.01) is associated with a trend in increasing risk of BC mortality. Relative to women who had a single childbirth, the adjusted HRs were 1.17 (95% CI = 0.51–2.69) for women who gave birth to two children, and 1.31 (95% CI = 0.56–3.10) for women with three or more childbirths, respectively. These results were not statistically significant. Study results suggests that giving birth at an early age may confer a protective effect on the risk of death from BC.

## 1. Introduction

In Taiwan, bladder cancer (BC) ranked 12th and 11th among leading causes of cancer mortality for men and women, respectively [1]. The age-adjusted mortality rate for BC in 2011 was 3.2 per 100,000 among men and 1.5 among women. Substantial geographic variation in BC mortality rates exists [2], with relatively high BC mortality in the urban areas and generally lower in rural Taiwan.

Tobacco smoking and exposure to industrial chemicals at work are established risk factors for BC [3,4,5,6,7]. The incidence of BC in men is approximately double to quadruple the rate in women [4,8,9,10,11]. However, the established risk factors do not entirely account for the gender difference in incidence [12,13]. Prior studies suggest that hormonal factors may explain in part the reduced risk of BC in women [14,15,16]. The protective effect of sex hormones on BC risk is theoretically possible given that estrogen receptors exist in normal female bladder tissue, bladder cell carcinomas, and bladder tumors [17,18,19,20].

Substantial epidemiologic evidence supports the hypothesis that endogenous hormones affect the etiology of several types of neoplasms, especially for cancers such as cancers of the breast and ovaries, which are hormone-dependent. Ceteris paribus, parous women have reduced risks of breast and ovarian cancers. The younger the age at first birth, the lower a woman’s risk is for developing these cancers, but the association between age and risk is complex [10]. There is only limited empirical evidence on the role of parity and age at first birth in the etiology of BC, and the results in the literature have been mixed. Some studies found an inverse association between higher parity and risk of BC [11,13], while others reported no association [7,15,16,21,22,23,24,25]. Likewise, studies have reported inconsistent results with respect to the relationship between late age at first birth and BC risk, with two studies showing decreased association [11,13] and others finding none [7,15,16,21,22,25,26]. The above-mentioned epidemiologic studies have been conducted primarily in America and some European countries.

Limited data exist as to the relationship between BC and reproductive factors [7,11,13,15,16,21,22,23,24,25,26]. Moreover, study results that do exist have not been consistent. To address the gap in studies exploring the association between parity, age at first birth, and the risk of BC, we studied a cohort of Taiwanese women who had a first and singleton childbirth between 1 January 1978 and 31 December 1987.

## 2. Materials and Methods

### 2.1. Data Source

Taiwan law requires the registration of births. Within 15 days of the childbirth, parents or the family must register infant births at a local household registration office. Since 1978, data on all live births are released by Taiwan’s Birth Registration System, which is managed by the Department of Interior. Physicians attending the birth complete the live-birth registration form, which includes information on maternal age, education, parity, gestational age, date of delivery, infant gender, and birth weight. Most deliveries in Taiwan occur in either a hospital or clinic [27]. As a result, Taiwan’s birth registration data are considered complete, reliable, and accurate. We have extensive experience using this data set [28,29]. This study was approved by the institutional review board of the Kaohsiung Medical University Hospital (KMUH-IRB-20130042).

### 2.2. Study Population

**Figure 1:**
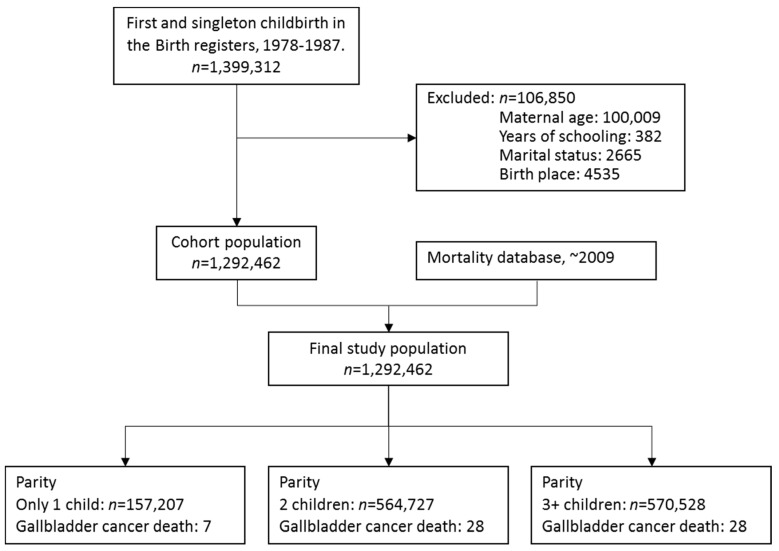
Flowchart of study subject selection.

All women with a first and singleton childbirth in the Birth Register between 1 January 1978 and 31 December 1987 are included in our cohort. During this period, a total of 1,399,312 first and singleton births in Taiwan were recorded. The Birth Registry also provided information on any subsequent births. Among the 1,399,312 primiparous women, we excluded 106,850 subjects because data were missing on one or more variables such as maternal age (*n* = 100,099), years of schooling (*n* = 382), marital status (*n* = 2665), or birth place (*n* = 4535). After removing data with missing observations, we were left with 1,292,462 women with complete information for the analysis (Figure 1). We have previously described the details of our cohort in an earlier publication [30].

### 2.3. Follow-Up

Every woman in our cohort was identified by a unique personal identification number (ID). Using this ID, we tracked the subject from the time of her first childbirth to 31 December 2009, and her vital status was verified via linking with the computerized mortality database, which permitted us to identify the date of any deaths. No subjects had a missing ID number among the 1,292,462 women followed, so we were able to follow all cohort members over time. Because the registration of deaths at local household registration offices is also mandated by law, the death statistics in Taiwan are considered to be highly accurate and complete [30,31].

### 2.4. Statistics

We calculated the person-years of follow-up for each subject from the date of first childbirth to the date of death or 31 December 2009. We calculated mortality rates by dividing the number of BC deaths by the number of person-years of follow-up. Cox proportional hazard regression models with time-varying covariates were used to estimate the hazard ratios (HRs) of BC death associated with parity (the total number of children recorded in the last childbirth record for each subject) and age at first birth. We also calculated the 95% confidence intervals (CIs) for the HRs. A cause of death resulting from primary BC is defined using the International Classification of Disease, Injury, and Causes of Death (9th revision) (ICD code 188). Age at first birth was divided into tertiles based on the entire cohort (<23, 23–26, or >26). Our final model included the variables age at first birth, parity (1, 2, 3 or more), marital status (married, unmarried), years of schooling (≤9, >9 years), and birth place (hospital/clinic, home/other). Both years of schooling and birth place were used as surrogate measures for socioeconomic status. We assessed the proportional hazards assumption for all of the aforementioned variables, and detected no violations. To test for trends in BC mortality risk with increasing levels (parity and age at first birth), we assigned ordinal numbers to the categorical variables for parity and median for age at first birth, and estimated our risk models with these variables as continuous variables. The statistical significance of the corresponding coefficient were assessed using the Wald test [32]. Analyses were performed using the SAS statistical package (version 9.2, SAS Institute Inc., Cary, NC, USA). All statistical tests were two-sided at conventional levels of statistical significance (*p* values < 0.05).

## 3. Results

A total of 1,292,462 primiparous women with complete information were included. Our selection criteria resulted in 34,980,246 person-years during the follow-up period from the time of first childbirth to 31 December 2009. We observed 63 BC deaths, representing a mortality rate of 0.18 cases per 100,000 person-years. None of the 63 BC deaths resided in a township where black foot disease was endemic (a surrogate of high arsenic exposure).

In Table 1, we present the numbers of person-years of follow-up and BC deaths categorized by age at first birth, parity, marital status, years of schooling, and birth place. The mortality rate was 0.17 per 100,000 person-years among women with one child, 0.19 per 100,000 person-years among those with two children, and 0.18 per 100,000 person-years among those who with 3 or more childbirths.

Table 2 shows the multivariate-adjusted HR and 95% CIs. We found an increased risk of BC with older age at first birth. Relative to women who gave birth at less than 23 years, the adjusted HR was 1.24 (95% CI = 0.66–2.35) for women who delivered between 23 and 26 years of age, and 2.30 (95% CI = 1.21–4.39) for women who delivered after 26 years of age. A trend of increasing BC mortality risk was detected with increasing age at first birth (*p* for trend = 0.01).

The HRs adjusted for age at first birth were 1.17 (95% CI = 0.51–2.69) for women who gave birth to two children, and 1.31 (95% CI = 0.56–3.10) for women who gave birth to three or more children, respectively, relative to women who gave birth to only one child. Our results suggest that increasing parity appears to be moderately associated with an increased risk of BC death. However, these results were not statistically significant, and there was no clear trend with parity (*p* = 0.58). This may be due to the relatively small number of BC deaths.

## 4. Discussion

The role of reproductive factors has not been studied extensively in BC research [7,11,13,15,16,21,22,23,24,25,26]. In our study, we found that BC mortality risk increased with greater age at first birth after adjusting for parity. Our finding that age at first birth is positively associated BC risk is consistent with two previous studies [11,13], but contrasts with other studies that reported no such association [7,15,16,21,22,25,26]. We do not know the cause for such discrepancies. Possible reasons include the following. First, it may be possible that an earlier age at first birth played a protective role in BC risk via elevated levels of certain hormones (such as estrogen) during pregnancy. Furthermore, a previous study has found that age at menarche (and therefore exposure to estrogens stimulation) is related to age at first birth [33]. Earlier increased exposure to estrogens from earlier regular menstrual cycles may protect against tumor initiation during this early stage of life. Because estrogens have been demonstrated to inhibit BC growth and development, findings from animal studies support a role for sex hormones in the etiology of BC [34,35,36]. Our finding that BC risk is positively associated with older age at first birth is consistent with the hypothesis that estrogen exposure is protective against BC risk. However, it is possible that our result is a chance finding because of the lack of consistent evidence to date for an association between age at first birth and BC mortality risk. Further empirical analysis will be required to establish a clear connection between age at first birth on the risk of BC.

Serum estrogen levels are elevated approximately 100-fold during pregnancy [37]. Greater parity increases the lifetime exposure to sex hormones. Animal studies support a direct role for sex hormones in the development and progression of BC because estrogens have been shown to inhibit BC [33,34,35], and because parous women have significantly smaller bladder tumors than nulliparous females [38]. Thus, it is possible that pregnancy offers some protection against BC if estrogens are associated with a reduced BC risk. Two prior studies reported reduced BC risk for parous women relative to nulliparous women [13,26], and additional studies examining parity and BC risk are consistent with a lower risk, but most did not achieve statistical significance [7,15,16,21,22,23,24,25] with the exception of the study by Weibull and colleagues. The first birth appears to account for most or all reduction in risk, as risk does not seem to depend on the number of births thereafter [25]. To our knowledge, ours is the sole study to report a possible increase in BC risk associated with greater number of births, although our finding has no statistical significance. Our results await future research for confirmation or refutation.

To date, only four prospective studies have investigated the association between parity, age at first birth, and BC risk [11,15,22,23]. The existing literature primarily used a case-control design [7,13,21,24,25,26]. The prospective study design is a main strength of our study, as it removes the possibility of recall bias. In addition, the use of virtually all available study subjects in a population and their follow-up eliminates selection bias in the study. The mean age at baseline was 24.33 years, therefore the subjects are younger than the studied in previous cohort analyses (ranging from 30 to 69) [15,22,23]. Furthermore, age at first birth may be different in different countries and during different periods. The mean age at first birth was 24.33 in our cohort. This value was increased during the study period (ranged from 23.3 to 28.1 in 2006). Likewise, the same temporal differences may exist for European countries. The mean age at death for BC was 47.32 ± 5.66 years in this study. The majority of BC deaths (79.37%) were premenopausal (using age 52 as the cut-off value), i.e., they were younger than subjects studied in previous studies (age at BC diagnosed ranging from 55 to 86). The mean time of BC death was about 17.37 years after last delivery (age at the birth of the last child = 29.95 ± 4.25; age at the death for BC = 47.32 ± 5.66). The mean age at last follow-up was 51.64 ± 3.84, which is also lower than the mean age of subjects in previous studies (ranging from 70.6 to 80). Our mean follow-up period was 27.06 (standard deviation is 3.35) years. Our finding is particularly noteworthy because women included in this study were young (the majority of BC deaths occurred before premenopause) and were among the youngest reported in any study.

A prerequisite for drawing any conclusion from mortality analysis is an assessment of the completeness and accuracy of the death registration system. In Taiwan, a decedent’s family is required to obtain a death certificate from the hospital or local community clinic and submitted it to the local household registration office. The death certificate which must be completed by a physician in Taiwan is required for the decedent to be buried or cremated. Therefore, the death registration in Taiwan can be considered reliable and complete. Data on the accuracy of BC diagnosis are not available in Taiwan and misclassification is possible. However, this misclassification is likely to be nondifferential (i.e., unlikely to be related to parity and age at first birth) and therefore would tend to underestimate rather than overestimate the true association. Access to population-level data and follow-up made possible by the national ID made it likely that selection bias is not a significant confounder in our study. Information bias is also not likely to be significant for parity and age at first birth.

In 1995, Taiwan implemented its National Health Insurance (NHI) program, which covers healthcare needs for all citizens of Taiwan. By 2007, the NHI program provided coverage for 22.60 million out of 22.96 million Taiwanese citizens (coverage rate of 98.4%) [39]. Taiwan has a convenient communication network and is only slightly larger than the U.S. state of Maryland. It is therefore feasible that all BC patients had access to medical care and would therefore be included in our data. The overall five-year survival rate of BC is approaching 80% [10]. However, there is no evidence to suggest that parity, age at first birth, and the five-year survival rate of BC is correlated. We therefore expect that the use of mortality data rather than observations on inpatient admissions would not have a confounding effect on the associations that we observed.

Several limitations of this study must be noted. First, Taiwan’s vital statistic and birth registration system records only live births and not stillbirths and abortions. It was therefore not possible for us to examine the potential influence of gravidity on BC risk. Secondly, our study focused, by design, solely on mortality among women who had given births, and we were not able to examine the possible role of nulliparity on the risk of BC. Our findings therefore have limited generalizability. Third, two studies have reported that hormone replacement therapy (HRT) was associated with an increased risk of BC [7,40]. Other studies, however, have failed to uncover a statistically significant association between HRT and BC [15,16,22,24,25]. With respect to the use of oral contraceptives (OCs), previous studies have largely reported no association between their use and BC risk [7,15,16,22,24]. We were unable to control for these two hormonal factors due to the lack of data. Nevertheless, the use of OCs and HRT is low in Taiwan compared with Western countries [41,42], so the omitted variable bias from these factors should be small, if any. The limited exposure to OCs and HRT allowed us to disentangle the impact of exogenous hormones from our assessment of parity and age at first birth. Fourth, smoking is consistently accepted as a causal risk factor for BC [4]. We did not control for smoking in our study, but the prevalence of tobacco smoking is only 4.2% among Taiwanese women [43]. Our study is therefore unlikely to be affected by cigarette smoking given its low prevalence. Fifth, women aged 65 years or older have the highest BC mortality rate in Taiwan. Since the study population of this study is relatively young in comparison (mean age at death for BC was 47.32 ± 5.66; mean age at last follow-up was 51.64 ± 3.84), and had not reached the age associated with the highest BC risk, the generalizability of our findings may be limited. Finally, unmeasured confounding remains a possible source of bias: our data did not include information on other potential occupational and environmental risk factors, such as hair dyes, aromatic amines, polyaromatic hydrocarbons, and diesel engine exhaust [10]. Age at first birth explains at least in part geographic and socioeconomic disparities, given that women with lower levels of education are considerably younger at first birth. In our study, we used years of schooling as a proxy for socioeconomic status, so we may have partially indirectly controlled for other unobserved occupational and environmental factors.

## 5. Conclusions

We conclude that increasing parity may be associated with a moderately increased risk of BC, even if the result was not statistically significant. We also found that BC risk was higher with greater age at first birth. Despite substantial experimental evidence linking hormonal factors with BC, epidemiologic evidence remains inconsistent. More research will be required to better understand the relationship between parity, age art first birth, and BC risk.

## Figures and Tables

**Table 1:** Demographic characteristics of the study cohort.

Variables	No. of Subjects	Follow-Up Person-Years	No. of Deaths from Bladder Cancer	Mortality Rate (per 100,000 Person-Years)
Age at recruitment (first birth)				
≤23	551,759	15,312,470.08	21	0.14
>23~≤26	433,114	11,592,980.92	19	0.16
>26	307,589	8,074,795.00	23	0.28
Parity (children)				
1	157,207	4,170,772.33	7	0.17
2	564,727	15,124,112.33	28	0.19
3+	570,528	15,685,361.33	28	0.18
Marital status				
Married	1,260,615	34,115,479.25	63	0.18
Not married	31,847	864,766.75	0	0.00
Years of schooling				
≤9 years	722,518	19,850,938.17	31	0.16
>9 years	569,944	15,129,307.83	32	0.21
Birth place				
Hospital/clinic	1,245,925	33,638,862.83	60	0.18
Home/other	46,537	1,341,383.17	3	0.22

**Table 2:** Association between parity, age at first birth, and relative risk of death from bladder cancer over a 32-year follow-up period.

Variables	Crude RR (95% CI)	Multivariate-Adjusted RR * (95% CI)
Age at recruitment (first birth)		
≤23	1.00	1.00
>23–26	1.29 (0.69–2.40)	1.24 (0.66–2.35)
>26	2.33 (1.29–4.22)	2.30 (1.21–4.39)
	*p = 0.01 for linear trend*	*p = 0.01 for linear trend*
Parity (children)		
1	1.00	1.00
2	1.10 (0.48–2.51)	1.17 (0.51–2.69)
3+	1.01 (0.44–2.31)	1.31 (0.56–3.10)
	*p = 0.89 for linear trend*	*p = 0.58 for linear trend*
Marital status		
Married	1.00	1.00
Not married	–	–
Years of schooling		
≤9 years	1.00	1.00
>9 years	1.46 (0.89–2.40)	1.25 (0.73–2.13)
Birth place		
Hospital/clinic	1.00	1.00
Home/other	1.11 (0.35–3.55)	1.35 (0.42–4.40)

* mutually adjusted; RR: relative risk; CI: confidence interval.
